# A scoping literature review of the associations between highly visual social media use and eating disorders and disordered eating: a changing landscape

**DOI:** 10.1186/s40337-023-00898-6

**Published:** 2023-09-26

**Authors:** Ashley Sharma, Carol Vidal

**Affiliations:** 1 Department of Mental Health, Johns Hopkins Bloomberg School of Public Health, 615 N Wolfe St, Baltimore, MD 21205 USA; 2 Division of Child and Adolescent Psychiatry, Department of Psychiatry and Behavioral Sciences, Johns Hopkins School of Medicine, 733 N Broadway, Baltimore, MD 21205 USA

**Keywords:** Eating disorders, Disordered eating, Body image, Highly visual social media, Gender

## Abstract

**Background:**

Although the etiology of eating disorders (ED) and disorder eating (DE) is multifactorial, exposure to highly visual social media (HVSM) may be an important contributor to the onset or worsening of DE and ED symptoms. We aim to understand HVSM use, ED, and DE with a particular focus on gender differences, as well as details of engagement on “selfies” in adolescents and young adults (AYA) through a scoping review of the literature.

**Methods:**

We conducted a literature search in Psycho ED, PubMed, MEDLINE of articles, including participants with DE/ED and users of HVSM, focused on AYA. Studies in which the study population did not include AYA, the SM platforms used did not include HVSM platforms, and the methodology to assess ED/HVSM use was not robust were excluded.

**Results:**

We found a strong relationship between HVSM and ED and DE with existing gender differences related to the nature of engagement and preference of content. The literature also shows effects of the specific mechanisms of use of these platforms involving “selfie” preparation and posting. Existing research is limited and consists of mostly cross-sectional studies with no uniform methodology and with participant populations that are not well-defined.

**Conclusions:**

The use of unregulated and profit-driven SM platforms can increase risk for ED. To use these HVSM platforms for positive influence, there is a need to have more transparency, and involvement of clinicians, researchers, and educators.

**Public significance:**

Due to HVSM’s popularity among the AYA population, it is important to identify its effects on the development of DE and ED, as well as recognize any gender differences. Clinicians, parents, and other adults working with youth should be aware of HVSM’s impact on DE/ED, as described in this review.

## Abstract (plain language summary)

We reviewed existing literature considering the effects of highly visual social media (HVSM) on the development of eating disorders (ED) and disordered eating (DE) in the adolescent and young adult (AYA) population. There are gender differences in the use of HVSM and its associations with DE/ED through females’ and males’ distinct engagement styles. The main individual drivers for DE/ED are worsened body image, low self-esteem, and low body dissatisfaction, and these are influenced by other individual, family, and social factors. In addition, maladaptive use of HVSM leads to tolerance, withdrawal, and craving and is linked to anxiety and depression. Total time spent on HVSM, preoccupation with photo editing, and posting with blurred demarcation of real vs. virtual presentations is associated with DE/ED development. Given HVSM’s popularity among the AYA and the risk for ED/DE in this population, it is important for clinicians, parents and other adults working with youth to be aware of HVSM’s impacts.

## Introduction

Since its advent, social media (SM) use has increased exponentially among adolescents [1]. More recently, highly-visual social media (HVSM), which are SM platforms that consist of sharing images or videos, have gained popularity. HVSM platforms, such as Instagram and Snapchat, allow for the editing, filtering, posting, sharing, and tagging of photos. The use of HVSM has been associated with body image concerns and disordered eating (DE) behaviors [2]. Simultaneously, the critical developmental period of adolescence [3] is characterized by an increased focus on body image, which is linked to both self and social worth, and is influenced by body weight and shape [4]. This confluence of adolescence and HVSM use can influence the prevalence and management of eating disorders (ED) and DE, especially among girls [5].

ED are associated with severe disturbances in people’s eating patterns and related emotions and thoughts, including preoccupation with food, body weight, and shape. ED include, but are not limited to, anorexia nervosa (AN), bulimia nervosa (BN), and binge eating disorder (BED) [6]. In their lifetime, 13% of adolescent girls and women experience a clinical or subclinical ED [7], and 60% actively try to lose weight despite being within the normal weight range. Males may have a higher drive for muscularity, which in extreme cases may lead to muscle dysmorphic disorder [8]. Across genders, a maladaptive focus on body image [9, 10] may cause body dissatisfaction, which has been identified as the most powerful predictor and risk factor for the development of DE [11].

DE and ED are among the most gendered of mental health disorders with strong associations with the female gender. Eating behavior associations with the female gender date back to the Middle Ages when fasting was associated with holiness, and have evolved with societal changes to a focus less on religion and more on body self-presentation [4]. Today, preoccupation with weight is such an integral part of the adolescent female experience that psychologists have coined the term “normative discontent’” to explain the idea that it is common to be unhappy with your weight if you are a female [12]. For example, in an Australian study with 67 girls focusing on the importance of body-image, many participants would prefer to be size 10 than get straight A’s [13].

HVSM has a complex interaction with ED and DE. Engagement on HVSM platforms through uploading visual media can transform contemporary everyday life into a “more photographic” life presented as a ‘selfie,’ which refers to a photograph of oneself that is typically shared through SM [14]. HVSM is used ubiquitously as a ready and primary source of information on food, exercise, and beauty standards with unprecedented access to advertisements and posts from peers, celebrities, and SM influencers [15], as well as personalized ad content. The engagement with content either as a producer, consumer, or prosumer (producer and consumer) promotes social comparison, the internalization of thin/muscular ideal, and the aim to adopt an observer’s perspective of their bodies and to habitually monitor themselves [16–19], contributing to the development and maintenance of DE and ED [20, 21]. Specifically, the internalization of the thin/muscular idea is influenced using SM filters. Filtered photo activity is positively associated with body dissatisfaction, and thin ideal internalization may mediate this relationship [22]. HVSM influences overvaluation of weight and shape, fear of weight gain, and preoccupation with weight and shape, contributing to core ED psychopathology [20, 23].

With regards to sex and gender differences, pubertal changes have differing effects on body image for females and males. Particularly, an increase in estrogen, which drives pubertal development in females, regulates gene transcription in neurotransmitter systems that are disrupted in ED (e.g., serotonin), and estrogen receptor 1 gene (ESR 1) has been associated with restrictive AN [24]. Furthermore, increase in testosterone, characteristic of pubertal development in males, seems to be a protective factor against DE [25–27]. Notably, there is more limited discussion regarding these topics in males since males are underrepresented in most studies. In an analysis of 20 peer reviewed articles on the use of SM on body image and DE, only eight studies had male representation [28].

Two recent meta-analysis, centering on SM before the popularization of the SM platform Tik Tok, focused on adult populations. A recent comprehensive meta-analysis inclusive of 127 studies showed that thin/athletic media exposure influences the internalization of thin ideal and ED across genders [9].

Instead of conducting a quantitative synthesis of research studies and literature pertaining to HVSM, DE, and ED, we sought to deliver a descriptive overview of this literature through a scoping review. In particular, with this scoping review, we aim to understand the key drivers that lead HVSM use to influence the development of ED/DE with a particular focus on gender differences. For this review, gender will be considered binary, as little relevant research has included individuals identifying as non-binary. Based on this review of the literature, we also aim to propose a theoretical Host-Environment model for DE/ED and HVSM use.

## Methods

### Search strategy

A search was performed in November 2022 in Psych Info, PubMed, OVID and EBCO databases using all combinations of the following terms: ‘social media’, ‘social networking sites’, ‘visual social media’ ‘Facebook’, ‘twitter’, ‘Instagram’, ‘Tumblr’, ‘Pinterest’, and ‘Flickr’, ‘TikTok’, ‘snapchat’ intersected with ‘eating disorder*’, ‘disordered eating’, ‘body image’, ‘self -esteem’, ‘body dissatisfaction’, ‘drive for thinness’, ‘drive for muscularity’, ‘fitspiration’ (a combination of “fit” and “inspiration”), ‘thin ideal’, ‘bulimia’, ‘anorexia’, ‘binge eating’, ‘orthorexia’, ‘selfie,’ ‘photo manipulation ‘, ‘posting ‘, ‘editing ‘, and ‘ gender.’ References of the collected articles were also scanned for additional relevant studies.

### Eligibility criteria

Studies and articles published between 2015 and 2022 that examined HVSM and its association with ED, DE and ED’s risk factors were analyzed.

### Inclusion criteria

Studies specifying the following criteria were included in the analysis: (1) The independent variable was exclusive or predominant use of HVSM; (2) The dependent variable was any of these terms: eating disorder; disordered eating; self-esteem; body image, body dissatisfaction, self -esteem, gender, time, selfie, photo manipulation, posting of selfies; (3) the study had a well-defined design and statistical analysis; (4) the manuscript was written in English and published between 2015 and 2022.

### Exclusion criteria

The following exclusion criteria were established: (1) presentations, dissertations, theses, books, book chapters, communications at conferences, and other technical documents; (2) mainly qualitative studies; (3) studies about contact and dating websites, chats, forums, online game pages, and virtual reality apps; (4) articles aimed at evaluating and examining SM groups that promote anorexia and bulimia; (5) articles on specific populations (e.g., athletes, dancers, models, and others), which could lead to bias in the data; and (6) validations of standardized tests and instruments.

The final studies analysis fulfilled the following PICOS statement (Table 1).

**Table 1:** PICOS statement

Participants:	Mean age < 25 years
Gender	Binary (male and female); non-binary not included
Intervention	Use of HVSM
Comparison	No engagement with HVSM vs. Engagement with HVSM
Outcomes	Effect on defined dependent variables of gender, time, processing of selfies in context of ED or DE

### Selection of studies for in-depth review

**Fig. 1:**
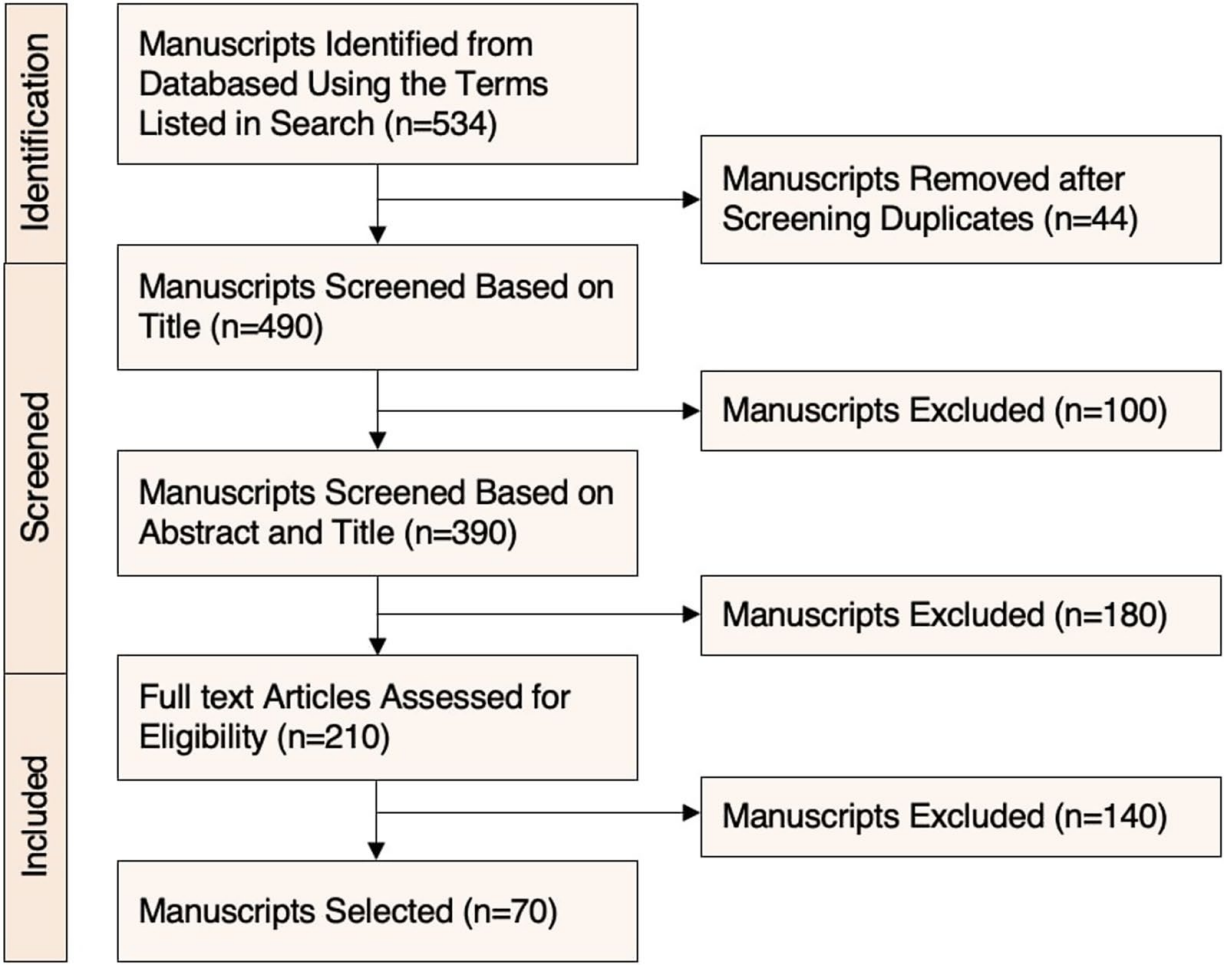
Flowchart of article selection

In compliance with the PRISMA like guidelines, a flowchart was used to provide a general overview of the article selection process [29]. The first author (A.S.) reviewed and screened articles sequentially by title, abstract, and full text review. The search yielded 534 articles which were screened, and 44 duplicates were removed. Of the 490 manuscripts screened based on the title, 100 total manuscripts were eliminated based on the inclusion criteria. The remaining 390 manuscripts were then screened based on abstract, which resulted in the exclusion of 180 manuscripts. Finally, 210 manuscripts assessment for eligibility after a full text review resulted in elimination of 140 manuscripts, and a total of 70 were selected for this literature review (Fig. 1).

Manuscripts were eliminated if the SM platforms included were not HVSM platform(s), if DE/ED or DE/ED symptomology were not mentioned, and if the study population did not include the AYA population. As full-text articles were being evaluated, 140 manuscripts were eliminated due to lack of (1) robust methodology, (2) inclusion criteria, (3) clear discussion of the relationship between HVSM and ED/DE.

A template to extract the following information was generated: (1) Author and year of publication; (2) Objectives of the study; (3) Study method; (4) Country of study population; (5) Demographic profile of the participants; (6) Results.

## Results

Early work on SM and ED focused primarily on the association between overall time spent on SM platforms and causal factors for ED, such as worsened body image, body dissatisfaction, and lower self-esteem [18]. However, current research recognizes the increasing role of appearance-based engagement on SM platforms [30] and the complex interactions with body image and body satisfaction [31].

This literature review findings are presented after a review of the existing literature surrounding HVSM and ED/DE, emphasizing time and mode of engagement, as well as gender differences regarding the use of HVSM sites.

The subsections are: (1) Time spent on HVSM, (2) Gender differences in frequency and mode of HVSM use, and (3) Gender differences on various selfie-related behavior/activities, such as (a) photo investment and manipulation, (b) posting a selfie, (c) viewing other selfies, and (d) viewing one’s selfies.

### Time spent on HVSM

There is robust evidence regarding the negative effects of HVSM on body image, depression, social comparison, and DE [28]. Weight and appearance-related esteem mediate the relationship between excessive time on SM and restrained eating across genders, and appearance-related esteem mediates the relationship between excessive time on SM and emotional eating for females [32].

Furthermore, literature indicates that higher levels of HVSM engagement, both in terms of frequency and volume, have been associated with an increased likelihood of experiencing eating-related concerns, such as DE, among adolescents [33]. This trend is accompanied by findings revealing that body dissatisfaction is associated with not only the extent of time spent on HVSM across genders but also with specific online behaviors, such as social comparisons and upward social comparisons with female peers [37, 38].

Further insights emerge from studies highlighting distinctions in how time on HVSM is spent between individuals with eating disorders (ED) and control groups. Despite similar overall online time, those with ED allocate more time to content centered around eating, weight, and body image on HVSM platforms compared to control counterparts [22, 39, 40].

In summary, time spent on HVSM significantly and positively correlates with measures of symptomatology of ED, such as body dissatisfaction, and negatively with measures of psychological health in both genders [33, 37, 38].

### Gender differences in HVSM use

The literature is equivocal on gender differences in HVSM users with ED/DE.

Females are more passive compared to males in their SM use, scrolling through accounts, posts, or images regarding weight loss, diet plans, and celebrity content [43]. Upward comparison is a mediator of body dissatisfaction in females [34]. On the other hand, research on male responses to HVSM content indicates that cognitive strategies, like critical evaluation and positive reframing are employed to navigate negative influences and choose positive content [42]. Yet, males show interest in content by athletes and bodybuilders, particularly fitspiration, which appears to shift their health concerns towards appearance [43, 44].

An experimental study delved into the impacts of exposure to fitspiration posts versus traditional messaging, self-compassion messaging, or image-only control [35]. In this context, self-compassion messaging demonstrated its capacity to yield positive outcomes primarily among females, while images devoid of associated text emerged as the optimal choice for promoting positive outcomes among males [35]. However, an alternative study with a similar experimental design diverged in findings, revealing that exposure to fitspiration images correlated with decreased body satisfaction, in contrast to the enhancing effect of self-compassion images on body satisfaction and appreciation across genders [45].

Overall, females tend to passively interact with HVSM content related to weight-loss and diet, whereas males actively interact with HVSM content about fitspiration [43, 44].

A few salient studies, selected based on citation index and characterizing gender differences in HVSM use, are tabulated in Table 2. All the studies were cross-sectional studies.

**Table 2:** Gender differences in HVSM use

Author (year)	Main HVSM	Site	Study population [N, Gender (M/F), M Age (SD)]	Methods	Main findings
Dhir (2016) [36]	Any HVSM	Norway	Adolescents (N = 968) M/F ratio = 398/570 Age (SD) = 16.96 (4.5) Young Adults (N = 1472) M/F ratio = 478/994 Age (SD) = 24.18 (3.5) Adults: (N = 1323) M/F ratio = 378/945 Age (SD) = 39.73 (5.66)	Survey	Females were more likely to take personal/group selfies, post personal selfies, crop photos, and use photographic filters compared to males
Tamplin (2018) [37]	Any HVSM	Australia	Young Adults (N = 374) M/F ratio = 187/187, Age (SD) = 23.7 (3.8)	Survey Measured state body satisfaction, SM literacy, internalization of appearance ideals, and trait UAC	Negative effect of ideal image exposure on body satisfaction observed across genders
Fatt (2019) [38]	Instagram	Australia	Adolescents and young adults (N = 118) M/F ratio = 118/0 Age (SD) = 19.43 (1.92)	Survey A 9-point Likert-type scale, Physical Appearance Comparison Scale-Revised, internalization Muscular subscale from the male version of SATAQ-4R, EMI-2, BESAA	Fitspiration is more closely related to appearance than to health in men
Lonergan (2019) [39]	Any HVSM	Australia	Adolescents and young adults (N = 184) M/F = 89/95 Age (SD) = M: 20.13 (3.43) F: 19.73 (3.48)	Survey	Manipulation and concern about selfies posted may be risk correlates for body dissatisfaction in men and women
Daniels (2020) [40]	Facebook	United States	Adolescents and young adults (N = 189) M/F ratio = 78/111 Age (SD) = 19.78 (1.89)	Observation Viewed a Facebook profile with either an objectified or a nonobjectified profile photo and evaluated the profile owner	Female participants liked the non-objectified profile and profile photo better than the objectified profile and profile photo
Rodgers (2020) [2]	Any HVSM	United States	College students (N = 170) M/F ratio = 85/85 Age = 22.2	Survey Reported on dating app use, body shame, surveillance, body satisfaction, media ideal internalization, and controllability beliefs related to weight/shape	Among males, frequent checking of dating apps was positively correlated with body shame and negatively with beliefs regarding weight/shape controllability. Few associations emerged among females
Rodgers (2020) [41]	Any HVSM	Australia	Adolescents (N = 681) M/F ratio = 333/348 Age (SD) = 12.76 (0.74)	Survey Completed a questionnaire assessing SMU, depression, self-esteem, BMI, muscular ideal internalization, AC, BD, DE, and muscle-building behaviors	Biopsychosocial frameworks are useful for conceptualizing relationships between SMU and body image, eating, and muscle building outcomes, preferentially in males
Koterba (2021) [42]	Any HVSM	United States	Young adults (N = 276) M/F ratio = 58/218 Age (SD) = 20.03 (1.81)	Survey (NPI-13) Selfies taken in the past week that included only themselves and the number that included others	Though no gender difference emerged for selfies taken alone, selfies featuring others were more common among women
Mahon (2021) [43]	Any HVSM	Ireland	Adolescents (N = 29) M/F ratio = 6/23 Age (SD) = 15.31 (0.47)	Focus groups	Boys exhibited greater positive agency over their bodies and SMU and tended to use more active coping styles than girls
Mayoh (2021) [44]	Any HVSM	United Kingdom	Adolescents and young adults (N = 1213) M/F ratio = 826/1175 Age range = 18–24 years	Web-based survey	Men were more likely to view content posted by athletes and actively use fitspiration as a source of inspiration to gain muscle. Women were more likely to view content related to weight loss/diet plans/celebrities’ content and passively use fitspiration as inspiration to lose weight
Schettino (2022) [45]	Instagram	Italy/ Portugal	Young adults (N = 340; Italy = 211; Portugal = 129) M/F ratio = 88/252 Age (SD) = 23.08 (3.46)	Survey Completed measures on selfie-sharing, selfie-manipulation, APC, internalization of beauty ideals, and shame for their bodies	Gender significantly moderates the effect of appearance comparison on body shame on Instagram, with a significant effect only for females
Simon (2022) [46]	Instagram	Philippines	Undergraduate students (N = 897) M/F ratio = 141/756 Age (SD) = 20.04 (1.29)	Survey	Physical appearance perfectionism significantly mediates the relationship between Instagram addiction and body esteem, with gender differences

ABC = Appearance based comparisons, AC = Appearance comparison Appearance Questionnaire-4 Revised, UAC = Upward appearance comparison, BESAA = Body Esteem Scale for Adolescents and Adults, BD = Body dissatisfaction, BMI = Body Mass Index, DE = Disordered Eating, EMI-2 = Exercise Motivation Inventory-2, SATAQ-4R = Sociocultural Attitudes Towards, SNS = Social Networking Sites, SM = Social Media, SMU = Social Media Use, NPI = Narcissistic Personality Inventory

## Gender differences in selfie-related behavior

### Photo investment and manipulation

Current evidence suggests that photo-centered activity, rather than total time spent on HVSM relates to adolescents’ body image disturbances [47]. In preparation to posting selfies, users typically engage in photo investment and photo manipulation [48], i.e. using filters [49]. This practice’s effect on ED causal factors is mixed [17].

Research signals to a “feedback loop” with photo investment, meaning that photo-editing is a consequence of body dissatisfaction, and body dissatisfaction further reinforces the behavior of editing [50]. This loop is driven by increased body surveillance and heightened awareness towards perceived flaws and imperfections [51].

While prior studies have found a positive relationship between photo investment and manipulation and the development of ED causal factors, such as body dissatisfaction [52], a few studies did not find a significant association between photo manipulation and body dissatisfaction [62, 63]. Indeed, some research suggests that editing photos without posting is associated with an immediate decrease in weight/shape concerns and a delayed decrease in sadness [62, 63, 66]. Therefore, existing research regarding impacts of photo investment and manipulation show mixed results.

### Posting a selfie on HVSM

Selfie posting is a social behavior related to attention-seeking, communication, and entertainment motivations [53]. Additionally, selfie posting and self-objectification are bidirectional, in that selfie posting may precede or result from appearance dissatisfaction [54]. Research findings indicate that a substantial percentage of individuals aged 18 to 24 engage in taking selfies with a majority actively sharing these images multiple times daily [68].

This form of engagement is two-edged. Research suggests a positive relationship between selfie posting and increased self-confidence in the short term [55]. Over the long-term, posting selfies appears to result in worsened mood and body image, and posting retouched selfies results in more harmful effects [55].

Though there is complex relationship of selfie posting with body image and ED, offline selfies (taking selfies that are not shared), known as body-checking, is related to greater ED symptom severity in most research [56].

### Viewing other’s selfies

Through HVSM, adolescents can view their peers’ idealized and edited photos. Viewing others’ photos on HVSM risks DE through increasing body dissatisfaction and appearance comparisons [2]. Adolescents high in trait social comparison may be especially vulnerable to the deleterious effects of viewing others’ photos on body image and satisfaction [57].

Furthermore, the newly developed comprehensive Social Media Appearance Preoccupation Scale (SMAP) whose subscales include Online Self-Presentation, Appearance-Related Online Activity, and Appearance Comparison, also substantiated associations with DE [58].

### Viewing one’s own selfies

Adolescents are also highly attuned to quantifiable metrics of peer approval in the form of likes, comments, friends, and followers [59]. Many friends on HVSM has been shown to positively correlate with body image concerns [60] and dieting [61]. In terms of likes, neuroimaging studies have demonstrated greater activation in the brain’s reward circuitry (e.g., the nucleus accumbens) when adolescents view photos that receive high numbers of “likes,” especially when these were their own photos, suggesting that quantifiable approval of one’s online self-presentation may be especially rewarding. Additionally, the number of likes has been found to influence female’s inclination to continue sharing objectifying selfies on HVSM [59].

Peer approval is communicated through comments on adolescents’ posts. Longitudinal evidence suggests that HVSM use is generally associated with more appearance-related peer comments on adolescents’ SM posts [62]. Though the same study found that peer appearance-related comments are unrelated to body dissatisfaction, positive appearance related comments (compliments) have been implicated in adolescent girls’ self-objectification [63]. However, negative appearance related comments may be linked to adolescent females’ lower self-esteem and depression and to males’ tendency to act out [64].

In summary, the literature shows that each stage of the process of the selfie, including preparation, posting, and viewing, can be associated with risk factors for ED, such as negative effects on self-esteem, body satisfaction, and body image.

**Fig. 2:**
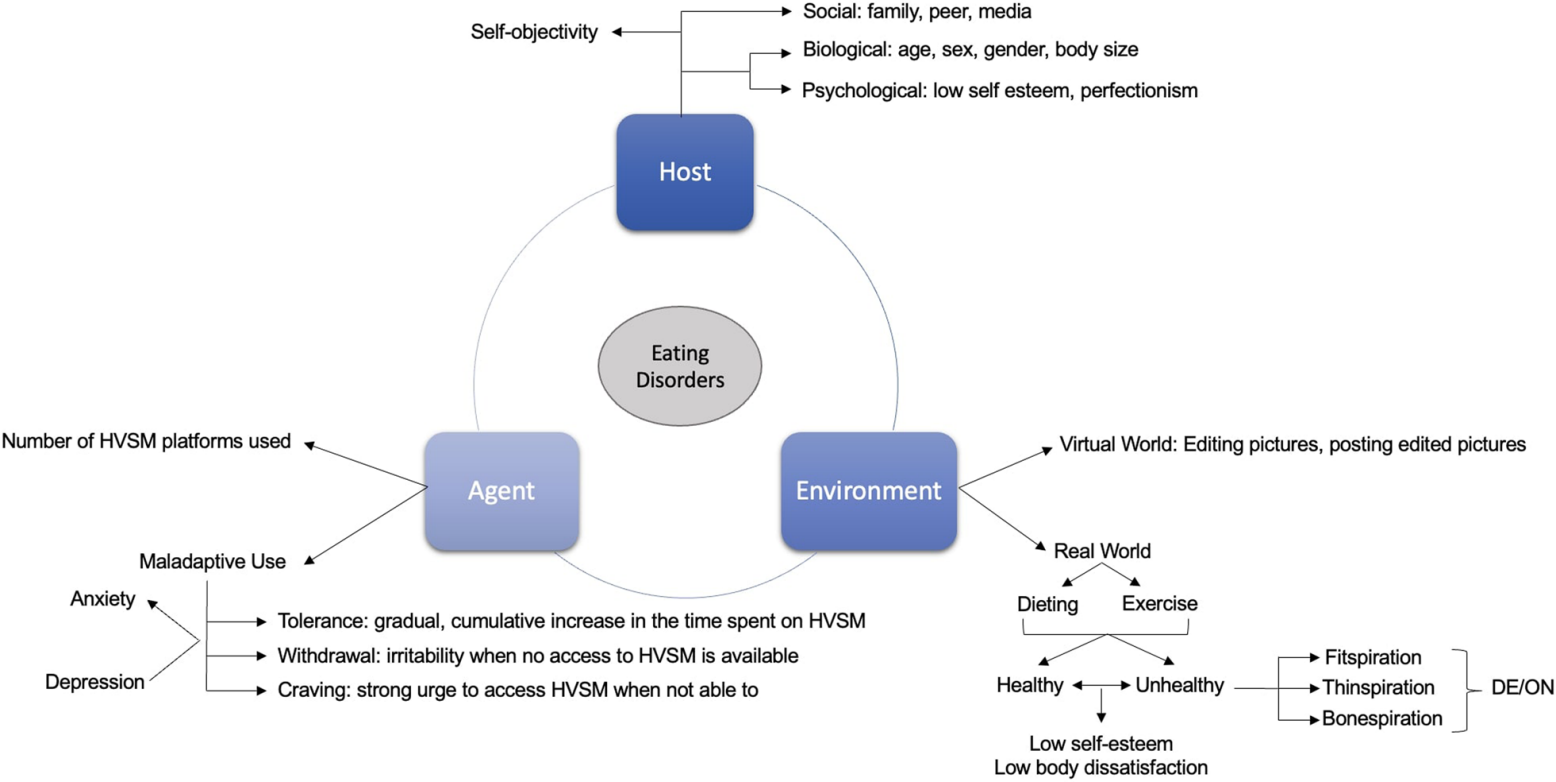
Agent-host-environment triad model adapted to eating disorders and highly visual SM use

Based on these results, we propose a model of ED/DE through a triangular, dynamic relationship between the host (individual with ED/DE), agent (HVSM use and engagement) and environment (food intake, exercise, edited selfies) that influence ED/DE presentations (Fig. 2). With regards to the host, there are a variety of biological (age, sex, and genetic predisposition) [65], psychological (low self-esteem, perfectionism) [18], and social factors (family, SM type, gender) that influence an individual’s susceptibility to develop ED. We hypothesize that the relationship between the individual predisposing factors and the development of ED may be linked to HVSM use, namely the number of HVSM platforms used and their maladaptive use, because HVSM use is linked to the development of recognized causal factors for ED, such as low self-esteem and body dissatisfaction [66]. Environments where practices, such as dieting and exercising are prevalent in the real world, and processes, such as editing selfies and posting edited-selfies are common on HVSM platforms, individuals’ inclination to use HVSM increases. In summary, it is the interaction of HVSM use and the individual’s engagement with the real and virtual world that affects the development or worsening of ED symptoms in individuals at risk.

## Discussion

This literature review, focusing on gender differences in highly visual social media (HVSM) engagement, unveils a complex interplay between DE, ED, the critical developmental phase of adolescence, and HVSM use. Notably, gender-specific tendencies in HVSM engagement surface with females displaying a more passive type of engagement and males more actively engaging with content on HVSM. Furthermore, the content sought out in HVSM differs by gender as well, as females gravitate toward dieting and weight-loss content, while males are drawn to fitspiration-oriented content. Importantly, despite these gender distinctions, the accumulated literature indicates that HVSM use does not singularly influence the development of DE/ED in females or males.

Delving further into the realm of HVSM's influence on DE/ED, a comprehensive summary derived from the analysis of 74 included articles reveals multifaceted dynamics. Photo-centered activities on HVSM, rather than mere time spent, appear to be associated to adolescents' body image disturbances [58]. In the context of selfie posting, a common HVSM behavior, a dual-edged impact unfolds. Short-term boosts in self-confidence are observed, but the long-term consequences suggest a decline in mood and worsened body image, particularly with retouched selfies [69]. This finding has also been shown in a recent comprehensive review (22 studies), where while nearly half (n = 10) of the included studies found an association between photo-editing and body-image, 4 reported mixed results, and 3 highlighted indirect associations between photo-editing and body-image concerns that were influenced by several constructs, including rumination and self-objectification [19]. Interestingly, offline selfies, indicative of bodychecking, align with greater ED symptom severity [57]. Viewing others' selfies on HVSM introduces the risk of increased body dissatisfaction and appearance comparisons, especially among those high in trait social comparison [58]. The comprehensive Social Media Appearance Preoccupation Scale (SMAP) corroborates associations between HVSM-related factors and DE [59]. Likes, comments, friends, and followers on HVSM serve as metrics of peer approval with varying effects. While many friends are linked to body image concerns and dieting, neuroimaging studies highlight the rewarding nature of likes, influencing self-presentation and affecting self-esteem [60]. Peer comments, whether positive or negative, exert diverse effects on adolescents' self-esteem, self-objectification, and mood [64, 65]. Each stage of the selfie process, encompassing preparation, posting, and viewing, intertwines with risk factors for ED, affecting self-esteem, body satisfaction, and body image.

As the research shows, extensive HVSM use contributes to risk factors of DE/ED, such as worsened body image and lowered body satisfaction, leading to DE and orthorexia nervosa, which refers to an obsession with healthy eating, which may evolve into ED [21]. In the case of females, because the advertised female body is unattainable for most women, their discouragement, captured through negative body image and decreased body satisfaction, and goal to achieve the ideal body type may lead them to practice DE to bridge the gap [67]. Both upward [68] and downward comparisons reinforce the behavior of HVSM use and engagement, contributing to eating behaviors, thereby suggesting a positive-feedback cycle and bidirectional relationship between HVSM engagement and DE/ED.

However, there is also potential of HVSM to be used as a therapeutic tool for treatment of DE/ED as shown in a feasibility study, which tested the efficacy of an intervention focused on appearance-related social media use on young adults, who are at heightened risk of developing an ED [69].

The results of this literature review have informed our proposed model which underscores a dynamic interplay between individual predisposing factors, HVSM use and engagement, and real-world and virtual environmental factors, forming a triangular bidirectional relationship influencing the development and exacerbation of ED symptoms in individuals with predisposing risks (Fig. 2).

This review has a few limitations. Of the studies reviewed, most were cross-sectional studies, which prevent us from making conclusions about causality and limits our conclusions to general associations between HVSM use and ED and DE. Most studies on HVSM quantity and quality of use (selfie behavior) collapse all HVSM and subjects into a single category; however, there are significant differences that must be accounted for, such as specific SM platform and gender. Furthermore, males are underrepresented in most of the studies. The evolving body ideals, coupled with gender-specific factors like body objectification and neurobiology, emphasize the gendered nature of ED/DE. Thus, research should encompass diverse biological, psychological, and cultural profiles, examining the interplay between HVSM and ED/DE causal factors after accounting for these potential confounding variables.

## Conclusions

In conclusion, the relationship between HVSM use and DE/ED is complex. Heightened emotional and social sensitivity, self-consciousness, and self-esteem influenced by HVSM engagement converge in influencing the emergence of DE/ED in adolescence. However, there HVSM may be potentially used as a therapeutic tool. This literature review demonstrates an unmet need for longitudinal and randomized control trials to understand and address differences across gender, age, sociocultural, other psychosocial individual, and family determinants to better define the relationship between HVSM use and DE/ED.

## Data Availability

Data sharing is not applicable to this article as no datasets were generated or analyzed during the current study.
